# Warm autoimmune haemolytic anaemia complicated by simultaneous arterial and venous thromboses: a case report

**DOI:** 10.11604/pamj.2022.42.260.25503

**Published:** 2022-08-08

**Authors:** Ela Ćurčić, Ozrenka Zlopaša, Sara Šundalić, Mia Rora, Radovan Radonić, Ana Vujaklija Brajković

**Affiliations:** 1 Division of Intensive Care Medicine, Department of Internal Medicine, University Hospital Centre Zagreb, Kišpatićeva 12, 10 000 Zagreb, Croatia

**Keywords:** Warm autoimmune haemolytic anaemia, thrombosis, anticoagulation, case report

## Abstract

The novelty described in this case report is the simultaneous development of arterial and venous thrombosis in a previously healthy Caucasian 37-year-old male with newly diagnosed warm autoimmune haemolytic anaemia (WA-AIHA). Clinical presentation included sensorimotor dysphasia, right arm paresis, abdominal pain, and swelling of the left leg. Computed tomography angiography showed partial occlusion of the left middle cerebral artery and multiple infarcts of the kidneys and spleen, while Doppler ultrasound revealed thrombosis of the left popliteal vein. A therapeutic dose of low-molecular-weight-heparin was instituted together with rituximab, leading to the complete serological and haematological remission. The exact thrombotic risk factors in WA-AIHA are still not completely identified and no generally accepted guidelines on thromboprophylaxis exist. The severe onset of the WA-AIHA might point towards a close association between haemolysis itself and thrombosis, raising the question of the necessity of prophylactic anticoagulation.

## Introduction

Warm autoimmune haemolytic anaemia (WA-AIHA) is a rare disorder caused by warm autoantibodies directed towards specific epitopes on the erythrocyte membrane, which can be a primary (idiopathic) disease, drug-induced or occur based on another underlying condition (in the majority of cases malignancies and connective tissue disorders) [1]. The overlap between AIHA and antiphospholipid syndrome (APS) is possible. Rottem *et al*. showed that AIHA occurred in 10.4% of patients with APS [2]. In that situation, arterial and/or venous thromboses are associated with the persistent presence of antiphospholipid antibodies. High-dose corticosteroids are the first-line therapy of the primary WA-AIHA, but there is still no generally accepted consensus as to whether splenectomy or rituximab should be the therapy of choice after the failure of the initial treatment with corticosteroids [3]. Venous thromboembolism (VTE), including pulmonary embolism as the most lethal complication [4], and less frequently thromboses of the arteries [5] have been reported in patients diagnosed with the disease, raising the question of whether patients with WA-AIHA should routinely receive prophylactic anticoagulation during disease exacerbations [3,5,6].

## Patient and observation

**Patient information and clinical findings:** a 37-year-old male with no prior medical history presented to the emergency department due to progressive fatigue, palpitations, and dark, cola-coloured urine, which he had noticed one week before admission.

**Diagnostic assessment and timeline:** laboratory workup revealed severe haemolytic anaemia (haemoglobin 5.8 g/dL, lactate dehydrogenase (LDH) 1962 U/L, indirect bilirubin 206 μmol/L, haptoglobin < 0.1 g/L). A peripheral blood film showed anisocytosis, polychromasia, and a few spherocytes. A direct antiglobulin test (DAT) was positive for IgG1, so the diagnosis of warm autoimmune haemolytic anaemia was established, and the patient was admitted to the general ward. Treatment with high-dose methylprednisolone was initiated in the general hospital. In the following days, the patient´s clinical condition deteriorated, with the development of severe abdominal pain, aphasia, and swelling of the left leg, due to which he was transferred to the medical intensive care unit of the tertiary teaching hospital.

**Figure 1:**
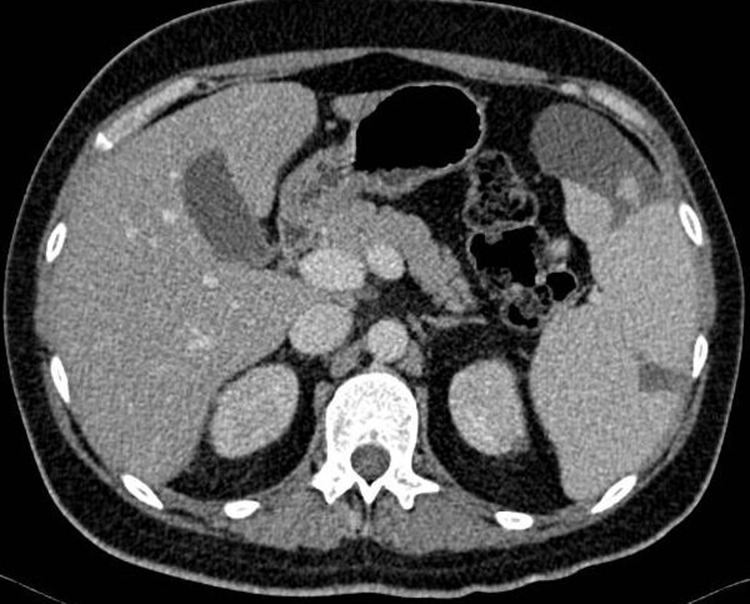
abdominal CT image showing multiple infarcts of the spleen

**Figure 2:**
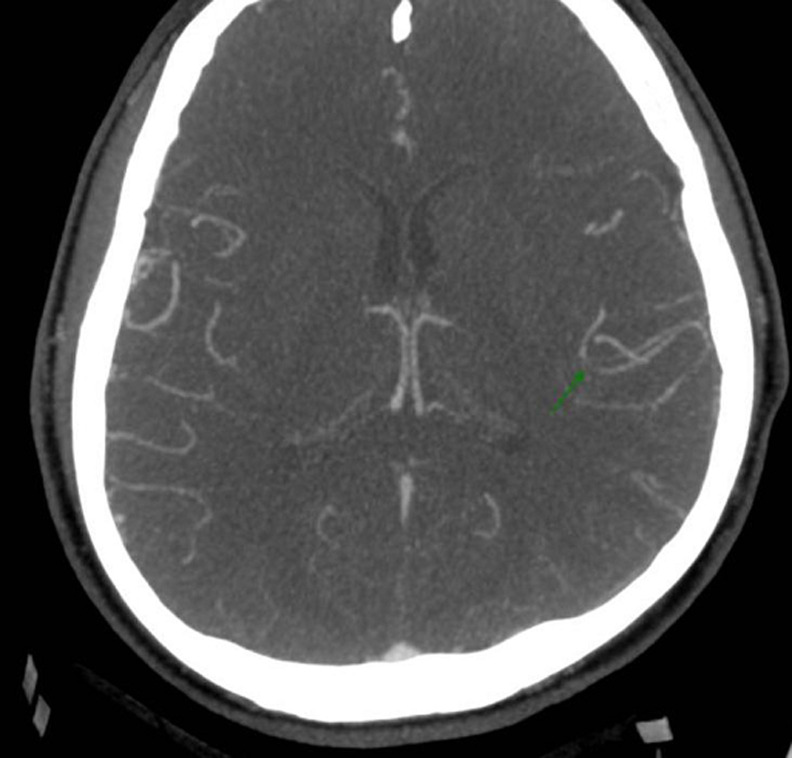
CT angiography image demonstrating partial occlusion of the left middle cerebral artery

Computed tomography of the abdomen showed multiple infarcts of the kidneys and spleen (Figure 1). The cardiac evaluation included echocardiography that revealed a structurally healthy heart, with preserved ventricular function and no signs of valvular disease, endocardial vegetations, or emboli. The neurological examination confirmed the diagnosis of sensorimotor dysphasia and right arm paresis. Computed tomography angiography showed partial occlusion of the left middle cerebral artery and concomitant ischemia of the parietal lobe, with no indication for endovascular treatment (Figure 2). Doppler ultrasound confirmed thrombosis of the left popliteal vein.

Additional testing was performed to eliminate other possible diagnoses. The possibility of drug-induced AIHA was excluded. Comprehensive laboratory work-up, including serum protein electrophoresis and immunofixation, antinuclear antibody (ANA), anti-neutrophil cytoplasmic autoantibody (ANCA), rheumatoid factor (RF), antiphospholipid antibodies (lupus anticoagulant - LA, anticardiolipin antibodies - aCL, anti-beta2-glycoprotein I antibodies - anti-B2GPI) and complement levels (C3, C4, CH50), was performed to rule out the potential underlying immunological disorder. Bone marrow biopsy and aspiration showed normal haematopoiesis, while computed tomography of thorax, abdomen, and pelvis excluded lymphoproliferative disease. Flow cytometry was performed to rule out paroxysmal nocturnal haemoglobinuria (PNH). Possible viral infection (EBV, CMV, hepatitis, HIV) was excluded from serology tests. Thrombophilia was also excluded based on the measurement of prothrombin time (PT), activated partial thromboplastin time (aPTT), fibrinogen, D-dimer, factor V Leiden, the prothrombin gene mutation, measurement of protein S, protein C, and antithrombin. Eventually, no underlying cause of WA-AIHA was found.

**Therapeutic intervention:** low-molecular-weight-heparin (LMWH) was instituted in the therapeutic dose and rituximab was introduced as a second-line therapy at a dose of 700 mg IV weekly for four weeks. The above resulted in clinical improvement with complete serological and haematological remission. The patient was discharged with a therapeutic dose of LMWH, acetylsalicylic acid, folate, vitamin B12, and methylprednisolone that was gradually tapered.

**Follow-up and outcomes:** the neurological deficit resolved after physical and logopedic therapy and two years after the initial presentation, the patient is well, working full-time, without any therapy.

**Patient perspective:** the treatment at the beginning of the disease was challenging. Despite initial treatment in the local hospital, my condition deteriorated. The worst complication was difficulty talking, abdominal pain, and weakness in the right arm. Everything happened quickly, which was even more shocking. The treatment was quite long. Prolonged physical therapy was necessary to overcome the neurological deficit. However, after two years I can lead a normal life.

**Informed consent:** the patient provided informed consent.

## Discussion

We report a rare case of a young male diagnosed with idiopathic WA-AIHA of severe onset and uncommon course of illness that was refractory to the recommended first-line treatment. The case represents an uncommon example of simultaneous arterial and venous thromboembolism in a previously healthy individual with no classical risk factors for VTE (calculated Padua Prediction Score 1), pointing towards a close association between haemolysis itself and its potential thromboembolic complications. Patients with AIHA have an increased risk of VTE [7]. It is presumed that multiple factors contribute to the development of VTE, including the presence of lupus anticoagulant (LA) [8] and toxic products of haemolysis [6,9], such as free haemoglobin, hem, and iron that induce a hypercoagulable state. Thrombotic complications in primary AIHA are more frequent in patients with severe disease onset (haemoglobin ≤ 8 g/dL, higher median LDH value), and previously splenectomized patients [10].

It is plausible that thrombotic risk associated with haemolysis is not only attributable to the classical risk factors considered important for the development of thrombosis in hospitalized patients (assessed with Padua Prediction Score). Our patient was young, and had a low Padua Prediction Score but had signs of severe haemolysis from the beginning of the disease which presumably made him more susceptible to the development of thrombotic complications. Having in mind that testing for LA can sometimes be falsely normal at the time of the acute phase of thrombosis (in contrast to the testing for aCL or anti-B2GPI antibodies), we repeated testing for antiphospholipid antibodies after several weeks. At both time intervals, the antiphospholipid antibodies were negative which implies that APS can be ruled out. Due to the rarity of the AIHA, the exact incidence of thrombotic events and risk factors for their occurrence are still not defined. Therefore, no generally accepted guidelines on thromboprophylaxis exist and the institution of prophylactic anticoagulation in this subset of patients is unfortunately still mainly clinician-, and not evidence-guided.

## Conclusion

This case report speaks in favour of prophylactic anticoagulation in patients with signs of severe haemolysis, regardless of their age and Padua Prediction Score. However, larger, multicenter studies are needed to define risk factors for thrombotic complications during haemolytic exacerbations to timely detect patients who may benefit most from prophylactic anticoagulation.
